# Editorial Notes and Comments

**Published:** 1911-11

**Authors:** 


### Editorial Notes and Comments.

The State Board of Health of Texas is issuing monthly a "Bulletin" that is really worth while. Ask for a copy free.

---

The Bloodless Phlebotomist.—We have received the October number of this journal, published by the Denver Chemical Co., N. Y. It contains a number of good articles of practical value and a copy may be had on request, mentioning the "Red Back."

---

Gray's Tonic is exceedingly grateful to take. When the weather is warm add a little cracked ice or ice water to a tablespoon of Gray's and you will get something better than a cocktail and which will do you much good without any injurious effects. There is no better restorative in convalescence from Grippe or any other exhausting ailment.

---

Solubility and Doses of Important Chemicals.—The Purdue Frederick Co., New York, have prepared a list of the more important drugs, giving dose, solubility in water at 25 degrees C. and in alcohol, 95 per cent, at 25 degrees C., which every doctor will find convenient for reference. It is a two-leaf folder. It can be had free on request.

IN THE QUIZ CLASS.—Prof. of Hygiene and State Med.: "What do you understand, Mr. Sporrer, by 'preventive medicine?'—give me an illustration."

Mr. Uppie T. Sporrer of Texas: "P-p-pr'ventive med'cin is —er-rer—med'cin what per-vents; like w-w-when un gives lead 'n opium f-f-fur the dier-ree! See?"

---

QUININE IN TRAUMATIC TETANUS.—Dr. W. W. Pugh, San Antonio, Texas, writes that he has treated successfully four cases of traumatic tetanus with sulphate quinine in "from 40 to 100 gr. doses, reducing the dose to 15 to 25 grs. every 4 to 6 hours, according to the effects of first dose." He reports the following case: "One case, Chas. Butler of Augusta, Texas, while lifting a post it slipped and a splinter penetrated the palmar parts of the hand about 3½ inches, and in 20 minutes he had spasms and was hardly able to get home. I at once gave him 75 grs. of quinine and the spasms ceased. Four hours later I had him take 40 grs. This is a treatment I have failed to report heretofore and am now doing so hoping that someone's life may receive the benefits thereof."

